# WHO consultation sets research priorities for monkeypox

**Published:** 2022-07

**3 June 2022 - Geneva -** A global research consultation convened by the WHO R&D Blueprint gathered over 500 experts and more than 2000 participants to discuss knowledge gaps and research priorities for monkeypox, in view of the recent outbreaks of the disease in both endemic and non-endemic countries.

Researchers and high-level experts from all over the world met virtually for two days to review the available evidence on the epidemiology of the virus; its transmission dynamics; the clinical characteristics; One Health research; community engagement; and countermeasures for managing the disease, including clinical care, treatments and vaccines. They agreed that effective countermeasures should be made available based on where the need was greatest.

Improved control of monkeypox in endemic countries is critical to address increases in disease incidence, and to control importations and outbreaks elsewhere. Participants agreed that strengthened collaboration among researchers in endemic countries, who have a wealth of experience and data on the disease - along with researchers from other countries - will ensure that scientific knowledge advances more quickly.

Experts underlined the need for expedited studies to better understand the disease epidemiology, its clinical consequences, and the role of various modes of transmission. In addition, the following research needs were highlighted: a comprehensive One Health approach to understand animal-to-human transmission and animal reservoirs; development and evaluation of better diagnostic tools that can be available around the world; improved approaches to communicate and engage communities in affected areas; studies to optimize supportive clinical care; documentation of the best control and treatment practices; and prompt and transparent communication of data and scientific evidence.

Experts also emphasized the need for clinical studies of vaccines and therapeutics to better document their efficacy and understand how to use them in this and future outbreaks.

Implementing without delay public health activities - such as communicating prevention information, enhanced disease surveillance, contact tracing, isolation of cases and optimized care of of people with the virus - should be used to limit spread and help the people affected, no matter where they are.

This consultation is part of a range of WHO activities in response to this multi-country outbreak.

**About the R&D Blueprint**

The R&D Blueprint is a global strategy and preparedness plan that allows the rapid activation of R&D activities during epidemics. Its aim is to fast-track the availability of effective tests, vaccines and medicines that can be used to save lives and avert large scale crisis.

For each of these diseases, R&D roadmaps and, where relevant, target product profiles (TPPs) and generic protocols are developed through broad and open consultations with leading experts and other stakeholders. In addition, efforts to strengthen national regulatory and ethics bodies to respond to public health emergencies are being implemented.

Available from: https://www.who.int/news/item/03-06-2022-who-consultations-sets-research-priorities-for-monkeypox

